# Stability analysis with general fuzzy measure: An application to social security organizations

**DOI:** 10.1371/journal.pone.0275594

**Published:** 2022-10-20

**Authors:** Nasim Arabjazi, Mohsen Rostamy-Malkhalifeh, Farhad Hosseinzadeh Lotfi, Mohammad Hasan Behzadi

**Affiliations:** Department of Mathematics, Faculty of Science, Science and Research Branch, Islamic Azad University, Tehran, Iran; University of Bonab, ISLAMIC REPUBLIC OF IRAN

## Abstract

An effective method for evaluating the efficiency of peer decision-making units (DMUs) is data envelope analysis (DEA). In engineering sciences and real-world management problems, uncertainty in input and output data always exists. To achieve reliable results, uncertainties must be taken into account. In this research, a General Fuzzy (*GF*) approach is designed to cope with uncertainty in the presence of fuzzy observations for categorizing and specifying stability radius and alterations ranges of efficient and inefficient DMUs, which is applicable to real-world decision-making problems. For this purpose, a DEA sensitivity analysis model is presented, which will be modeled by fuzzy sets. Then, by applying the General Fuzzy (*GF*) approach, the fuzzy DEA sensitivity analysis model is transformed into the equivalent crisp form of fuzzy chance constraints according to specific confidence levels. Finally, a numerical example and a case study of branches of the social security organization are presented to illustrate sensitivity and stability analysis in the presence of fuzzy data. The obtained results provide the input and output changes of the evaluated units according to the attitude and preference of the decision maker with different confidence levels so that the data changes in the fuzzy environment do not change the units’ classification from efficient to inefficient and vice versa.

## 1. Introduction

Scrutinizing the performance of entities and organizations is a crucial liability of the top-level management team that can be carried out by various techniques. Data envelopment analysis (DEA), first suggested by Charnes et al. [1] (Charnes-Cooper-Rhodes (CCR model)) and continued by Banker et al. [2] (Banker-Charnes-Cooper (BCC model)), is a nonparametric way to appraise the relative efficiency of decision-making units (DMUs) with multiple input-output. Both CCR and BCC have been developed for many topics and with many successful applications and case studies, as reported in the DEA published works [3–5]. Since DEA is data-based, data entry errors, statistical noise, and incomplete data create disturbances in evaluating DEA’s efficiency. A commonly asked question is, “How sensitive are the DMUs to possible variations in the data?” This matter has been considered as a performance stability analysis in DEA. Numerous studies have focused on this topic. For the most up-to-date sensitivity analysis problems, the interested reader can observe: Zhu [6], Jahanshahloo et al. [7], Abri et al. [8], Zamani and Borzouei [9], Neralić and Wendell [10], Hladík [11], Khoveyni and Eslami [12], Arabjazi et al. [13], among others.

In early studies of DEA stability analysis, which was named the “Algorithmic approach,” Charnes et al. [14] treated the sensitivity of a single output of an efficient unit by using the optimal basis matrix of an LP problem. This approach was extended and improved by a few sensitivity analysis papers by Charnes and Neralić in which sufficient conditions were determined to preserve efficiency (see, for example, [15, 16]).

Thompson et al. [17] and Thompson et al. [18] provided a DEA multiplier approach to sensitivity analysis using the strong complementary slackness condition (SCSC) of centrality. Sensitivity analysis based on the super-efficiency technique in DEA is also another type of sensitivity analysis in which the DMU under evaluation is not included in the reference set. Charnes et al. [19] and Charnes et al. [20] proposed a super-efficiency DEA model where the simultaneous proportional variation of all inputs and outputs is applied to a DMU under test. Seiford and Zhu [21, 22] considered input and output changes separately and proposed an iterative procedure to define an input stability region (ISR) and an output stability region (OSR). Zhu [6] presented the maximum radius that preserves efficiency or inefficiency when the data of all DMUs are changed simultaneously and unequally.

Metters et al. [23] inspected the stability of a DMU batch. They divided DMUs and then specified the stability of DMUs using a trial and error unit scheme. Jahanshahloo et al. [24] developed the largest stability region for BCC and additive models by supporting hyperplanes for the DMU under evaluation. Liu et al. [25] studied the sensitivity analysis for efficient DMU when the data uncertainty occurred locally. They considered the constancy of an efficient DMU by worsening a class of DMUs simultaneously in the same directions to keep the under evaluation DMU remaining on the efficient frontier. Boljunčić [26] used an iterative procedure so that data changes do not affect the optimal basis matrix, and parametric programming was applied to obtain possible input or output perturbation.

Mozaffari et al. [27] offereda step method(STEM), an interactive technique of multiple objective linear programming (MOLP), for maintaining efficiency classification when all interval data is simultaneously altered for the DMU under test. Abri et al. [28] to define the consistency radius for efficient DMUs and quasi-efficient DMUs presented a super-efficiency model in DEA. Singh [29] provided multiparametric sensitivity analysis by classifying the perturbation parameters as ‘‘focal” and ‘‘nonfocal” and found critical regions for the efficient DMU under evaluation. Wen et al. [30], Khalili-Damghani and Taghavifard [31] surveyed the stable states of efficiency of DMUs in a fuzzy environment. Hladik [32] proposed stability analysis in linear programming and addressed the expansion of the maximum tolerance area. Jahanshahloo et al. [7] obtained a stability region for inefficient DMUs. Their method was considered a new frontier with a performance score of *α* < 1 for a specific inefficient DMU that *α* defined as constant by the manager.

Abri [8] demonstrated the sensitivity of returns to scale by stability radius. Daneshvar et al. [33] offered a DEA model by modifying the variable returns to scale (VRS) model to improve the efficiency stability region obtained from this model. Khodabakhshi et al. [34] and He et al. [35] determined the stability radius based on input relaxation super-efficiency measure and interval data, respectively. Arabjazi et al. [36] studied efficiency stability in the presence of stochastic data for DMUs in which data perturbations do not affect their classifications.

Agarwal et al. [37] scrutinized the robustness of DEA efficiency scores by changing the reference set of the inefficient DMUs via a new slack model (NSM). Banker et al. [38] inspected stability in stochastic data envelopment analysis. They applied the stochastic DEA (SDEA) model and obtained the necessary and sufficient conditions to preserve the efficiency classification of all DMUs. Zamani and Borzouei [9] offered a tolerance area when an extra unit needs to be joined to the set of the observed units by defining hyperplanes of the production possibility set (*PPS*). Of late, an alternative method has been proposed, focusing on enlarging the maximum tolerance radius and tolerance region. See, for example, Ghazi et al. [39], Arabjazi et al. [13], and Neralić and Wendell [10].

Recently, chaotic systems have many potential applications in various fields of technical sciences and engineering. Among them, we can mention its applications in medical issues, financial issues, encryption techniques, control systems and optimization algorithms. Tian et al. [40] introduced a new deep-learned type-3 fuzzy logic system to develop a model for CO2 solubility estimation. Moreover, a new type-3 fuzzy logic system with an online optimization scheme is proposed for stabilizing and synchronizing financial chaotic systems [41]. For more information about the new type-3 fuzzy logic system, see references [42, 43].

Mombini et al. [44] focused on sensitivity analysis in DEA and proposed an approach to determine the stability radius of the cost efficiency of units with interval data. Mahla et al. [45] detailed the sensitivity and stability analysis of the fuzzy SBM DEA and obtained lower and upper sensitive bounds for input and output variables for both the inefficient and efficient DMUs to determine the input and output targets. In order to assess the efficacy of DMUs with uncertain random inputs and outputs, Jiang et al. [46] created an uncertain random DEA model. The sensitivity and stability of this new model were then examined in order to determine the stability radius of each DMU. Aslani Khiavi et al. [47] used the network inverse data envelopment analysis approach to develop a structure for the optimal regulation of the bullwhip effect and considered the effect of time and delays on the bullwhip effect score.

Also lately, innovative meta-heuristic algorithms with biological and naturalistic inspirations have been developed to address challenging real-world optimization problems. A prairie dog optimization algorithm was presented by Ezugwu et al. [48] and replicates the actions of prairie dogs in their natural environment. A dwarf mongoose optimization algorithm was suggested by Agushaka et al. [49] and mimics the foraging social interactions and ecological adaptations of three social groups of dwarf mongooses. A reptile search algorithm that is inspired by crocodile hunting behavior was also proposed by Abualigah et al. [50]. Moreover, Oyelade et al. [51] introduced the Ebola optimization search algorithm based on the spread mechanism of the Ebola virus disease. Readers can find references [52, 53] if they are interested in learning more about meta-heuristic techniques for optimization issues.

In standard models of DEA, it is supposed that the data values are known and fixed. However, in real conditions, there may be uncertainty and ambiguity. Many topics with many successful applications have examined situations in which the inputs and outputs are not exactly known. Recently, some researchers addressed the matter with fuzzy data [54–57]. Hence, the methods of sensitivity analysis proposed in DEA are not suitable for DMUs with fuzzy data. So in this paper, first, we present a DEA model in an uncertain environment, which will be modeled by fuzzy sets. Then, the sensitivity and stability analysis of the proposed fuzzy DEA modeling is also shown. To that end, by applying the General Fuzzy (*GF*) approach, the fuzzy DEA sensitivity analysis model is transformed into the equivalent crisp form of fuzzy chance constraints according to specific confidence levels.

The difference between our work and the existing fuzzy procedures is that the existing approaches consider the optimistic, pessimistic, and compromised attitudes of the decision maker based on the possibility, necessity, and credibility measures in three separate models in fuzzy DEA, while the proposed stability analysis takes into account the different optimistic-pessimistic attitudes of the decision maker in one model only by setting an adjustable parameter. The drawback of the possibility, necessity, and credibility measures is that these three measures reflect the decision maker’s attitude as extremely optimistic, pessimistic, and compromising. While in order to estimate the efficiency of DMUs, we require an adjustable, flexible, and applicable measure for fuzzy decision-making problems so that this measure can reflect different perspectives of the decision maker. To put it another way, in our proposed approach, decision makers can apply different attitudes that they have obtained according to their experiences and judgments by adjusting the optimistic-pessimistic parameter for better decision-making and forecasting. In addition, *GF* measure models are formulated in the form of fuzzy linear programming.

The main contributions of the current research can be mentioned as follows:

- Providing an additive DEA model with fuzzy data.
- Proposing a sensitivity and stability analysis of the fuzzy additive DEA model.
- Presenting novel general fuzzy approach and chance constrained programming to tackle the uncertainty of the fuzzy chance-constraints in the fuzzy DEA sensitivity analysis model and convert them into their equivalent crisp values.
- Determining the stability region and stability radius for both efficient and inefficient DMUs.
- The efficiency of the proposed approach is illustrated by a numerical example.
- Implementation of the suggested method in a real-world case study from social security organization.

The rest of the paper has been organized as follows: In Section 2, first the general fuzzy measure is introduced, and then some basic concepts about the DEA additive model with fuzzy data will be presented. A sensitivity and stability analysis technique for DMUs with fuzzy data is shown in Section 3. An illustrative numerical example and a case study of social security organizations are discussed in Section 4. In the final Section 5, conclusions are expressed.

## 2. Fundamentals and concepts

### 2.1. Mathematical notations and nomenclatures

The symbols, parameters, and variables that will be utilized in this study are defined as follows:

$$\begin{array}{cc} Indices & \begin{array}{cc} j & the\;index\;of\;DMUs,\;j=1,\ldots ,n\\ i & the\;index\;of\;inputs,\;i=1,\ldots ,m\\ r & the\;index\;of\;outputs,\;r=1,\ldots ,s\\ o & the\;index\;of\;DMU\;under\;test \end{array} \end{array}$$

$$\begin{array}{cc} Parameters & \begin{array}{cc} X_{j} & the\;inputs\;vector\;of\;DMU_{j}\\ Y_{j} & the\;outputs\;vector\;of\;DMU_{j}\\ x_{ij} & the\;i^{th}input\;of\;DMU_{j}\\ y_{rj} & the\;r^{th}output\;of\;DMU_{j}\\ X_{o} & the\;inputs\;vector\;of\;DMU_{o}under\;test\\ Y_{o} & the\;outputs\;vector\;of\;DMU_{o}under\;test\\ x_{io} & the\;i^{th}input\;of\;DMU_{o}under\;test\\ y_{ro} & the\;r^{th}output\;of\;DMU_{o}under\;test\\ {\tilde{\text{x}}}_{ij} & the\;i^{th}fuzzy\;input\;of\;DMU_{j}\\ {\tilde{\text{y}}}_{rj} & the\;r^{th}fuzzy\;output\;of\;DMU_{j}\\ {\tilde{\text{x}}}_{io} & the\;i^{th}fuzzy\;input\;of\;DMU_{o}under\;test\\ {\tilde{\text{y}}}_{ro} & the\;r^{th}fuzzy\;output\;of\;DMU_{o}under\;test\\ \tau & a\;crisp\;number\\ \gamma & a\;specific\;confidence\;level\;for\;satisfying\;the\;uncertain\;constraint\\ \eta & the\;optimistic-pessimistic\;parameter\;of\;general\;fuzzy\;\left(GF\right)\;measure\\ K & a\;large\;enough\;number \end{array} \end{array}$$

$$\begin{array}{cc} Variables & \begin{array}{cc} \mu_{j} & the\;non-negative\;coefficient\;of\;DMU_{j}\;data\;in\;the\;convex\;combination\\ s_{i}^{-} & non-radial\;decrease\;of\;i^{th}input\;of\;DMU_{o}\;under\;test\;in\;the\;additive\;model\\ s_{r}^{+} & non-radial\;increase\;of\;r^{th}output\;of\;DMU_{o}\;under\;test\;in\;the\;additive\;model\\ \rho_{i} & the\;stability\;radius\;of\;i^{th}input\;of\;DMU_{o}\;under\;test\\ \varphi_{r} & the\;stability\;radius\;of\;r^{th}output\;of\;DMU_{o}\;under\;test\\ \Omega & a\;binary\;variable\;for\;linearization\;of\;incompatible\;constraints \end{array} \end{array}$$

#### Definition 1

A region of permissible data changes is called a stability region of *DMU*_*o*_ if and only if the classification of all DMUs remains stable after such changes occur.

#### Definition 2

Given *DMU*_*o*_, the stability radius is the largest number, *r*, such that feasible perturbations to *DMU*_*o*_ strictly <*r* preserve the efficiency classification of all DMUs (Arabjazi et al. [13]).

### 2.2. General fuzzy measure

General Fuzzy (*GF*) is a measure used to express the chance of fuzzy phenomena that was developed by Xu and Zhou [58, 59]. Here, we will state some basic concepts in general fuzzy measure. For any fuzzy event *P* ∈ *Ψ*(Θ) that occurs on possibility space (Θ, Ψ(Θ), Pos), the general fuzzy measure is defined as follows:

GF{P}=Nec{P}+(η)(Pos{P}−Nec{P})=(η)Pos{P}+(1−η)Nec{P}

That is to say, the general fuzzy measure is equal to the convex combination of possibility (*Pos*) and necessity (*Nec*) in which *η* (0 ≤ *η* ≤ 1), is the optimistic-pessimistic parameter to determine the attitude of a decision-maker. *GF*{.} is a general fuzzy measure if and only if:

- *GF* {∅} = 0, *GF* {Θ} = 1
- ∀ *P* ∈ Ψ (Θ) ⇒ 0 ≤ *GF* {P} ≤ 1
- ∀ *P* ∈ Ψ (Θ), *η* ≤ 0.5 ⇒ 0 ≤ *GF* {*P*} + *GF* {*P*^*c*^} ≤ 1
- ∀ *P* ∈ Ψ (Θ), *η* ≥ 0.5 ⇒ 1 ≤ *GF* {*P*} + *GF* {*P*^*c*^} ≤ 2
- ∀ *P*, *R* ∈ Ψ (Θ), *P* ⊆ *R* ⇒ *GF* {*P*} ≤ *GF* {*R*}
- ∀ *P*, *R* ∈ Ψ (Θ), *η* ≥ 0.5 ⇒ *GF* {*P* ∪ *R*} ≤ *GF* {*P*} + *GF* {*R*}
- ∀ *P* ∈ Ψ (Θ) ⇒ *Pos* {P} ≥ *GF* {*P*} ≥ *Nec* {*P*}

Suppose that, $\tilde{\alpha }\left(\alpha_{1},\alpha_{2},\;\alpha_{3},\;\alpha_{4}\right)$ with the condition of *α*_1_ < *α*_2_ < *α*_3_, < *α*_4_ is a trapezoidal fuzzy variable on possibility space (Θ,Ψ(Θ), Pos). The membership function of $\tilde{\alpha }$ is expressed below:

$$\mu (x)=\left\{\begin{array}{ll} \frac{x-\alpha_{1}}{\alpha_{2}-\alpha_{1}}, & if\;\;\alpha_{1}\leq x\leq \alpha_{2};\\ 1, & if\;\;\alpha_{2}\leq x\leq \alpha_{3};\\ \frac{\alpha_{4}-x}{\alpha_{4}-\alpha_{3}}, & if\;\;\alpha_{3}\leq x\leq \alpha_{4};\\ 0, & otherwise. \end{array}\right.$$ (1)

Also, assume that *τ* is a crisp number. The *GF* measure of fuzzy events is as follows:

$$GF(\tilde{\alpha }\leq \tau )=\left\{\begin{array}{ll} 0, & if\;\;\alpha_{1}\geq \tau ;\\ \eta \left(\frac{\tau -\alpha_{1}}{\alpha_{2}-\alpha_{1}}\right), & if\;\;\alpha_{1}\leq \tau \leq \alpha_{2};\\ \eta , & if\;\;\alpha_{2}\leq \tau \leq \alpha_{3};\\ \eta +(1-\eta )\left(\frac{\tau -\alpha_{3}}{\alpha_{4}-\alpha_{3}}\right), & if\;\;\alpha_{3}\leq \tau \leq \alpha_{4};\\ 1, & if\;\;\alpha_{4}\leq \tau . \end{array}\right.$$ (2)

$$GF(\tilde{\alpha }\geq \tau )=\left\{\begin{array}{ll} 1, & if\;\;\alpha_{1}\geq \tau ;\\ \eta +(1-\eta )\left(\frac{\alpha_{2}-\tau }{\alpha_{2}-\alpha_{1}}\right), & if\;\;\alpha_{1}\leq \tau \leq \alpha_{2};\\ \eta , & if\;\;\alpha_{2}\leq \tau \leq \alpha_{3};\\ \eta \left(\frac{\alpha_{4}-\tau }{\alpha_{4}-\alpha_{3}}\right), & if\;\;\alpha_{3}\leq \tau \leq \alpha_{4};\\ 0, & if\;\;\alpha_{4}\leq \tau . \end{array}\right.$$ (3)

According to the fuzzy general measure properties, the certain counterparts crisp ones of fuzzy chance constraints under specific confidence level γ for both conditions of γ greater or less than η, are as follows:

$$GF\{\tilde{\alpha }\leq \tau \}\geq \gamma \Leftrightarrow \left\{\begin{array}{ll} \left(\frac{\eta -\gamma }{\eta }\right)\alpha_{1}+\left(\frac{\gamma }{\eta }\right)\alpha_{2}\leq \tau , & if\;\;\gamma \leq \eta ;\\ \left(\frac{1-\gamma }{1-\eta }\right)\alpha_{3}+\left(\frac{\gamma -\eta }{1-\eta }\right)\alpha_{4}\leq \tau , & if\;\;\gamma \geq \eta . \end{array}\right.$$ (4)

$$GF\{\tilde{\alpha }\geq \tau \}\geq \gamma \Leftrightarrow \left\{\begin{array}{ll} \left(\frac{\gamma }{\eta }\right)\alpha_{3}+\left(\frac{\eta -\gamma }{\eta }\right)\alpha_{4}\geq \tau , & if\;\;\gamma \leq \eta ;\\ \left(\frac{\gamma -\eta }{1-\eta }\right)\alpha_{1}+\left(\frac{1-\gamma }{1-\eta }\right)\alpha_{2}\geq \tau , & if\;\;\gamma \geq \eta . \end{array}\right.$$ (5)

In the above relations, if *η* is equal to 1, 0, and 0.5, then the *GF* measure represents possibility, necessity, and credibility, respectively [60, 61]. To put it another way, by setting *η* in the *GF* measure, the decision-maker can achieve the desired attitude. And lastly, in recent years, the popularity and applicability of the *GF* measure have been increasing among researchers, and it is widely used in real-world problems in fuzzy environments [62–64].

### 2.3. The DEA additive model

Assume that n DMUs need to be appraised. The symbols and notations are given as follows: DMU_*j*_ denotes the *j*th DMU, where *j* = 1, 2,*…*, *n*. DMU_o_ is the DMU under evaluation. The vectors *X*_*j*_ = (*x*_*1j*_, *x*_*2j*_,*…*, *x*_*mj*_) and *Y*_*j*_ = (*y*_*1j*_, *y*_*2j*_,*…*, *y*_*sj*_) are the inputs and outputs of DMU_*j*_,*j* = 1, 2, *…*, *n*. The inputs and outputs vectors of the target DMU_o_ are *X*_o_ = (*x*_1o_, *x*_2o_,*…*, *x*_mo_) and *Y*_o_ = (*y*_1o_, *y*_2o_,*…*, *y*_so_). Also, the production possibility set (*PPS*) and the additive model of Charnes et al. [14] are defined in Eqs (6) and (7) as follows:

$$PPS=\left\{\left(X,Y\right)\left|X\geq \sum_{j=1}^{n}\mu_{j}X_{j},Y\leq \sum_{j=1}^{n}\mu_{j}Y_{j},\sum_{j=1}^{n}\mu_{j}=1,\mu_{j}\geq 0,j=1,\ldots ,n\right.\right\}$$ (6)

$$\begin{array}{lll} Max & \sum_{i=1}^{m}s_{i}^{-}+\sum_{r=1}^{s}s_{r}^{+} & \\ s.t. & \sum_{j=1}^{n}\mu_{j}x_{ij}=x_{io}-s_{i}^{-} & \forall \;i\\ & \sum_{j=1}^{n}\mu_{j}y_{rj}=y_{ro}+s_{r}^{+} & \forall \;r\\ & \sum_{j=1}^{n}\mu_{j}=1 & \forall \;j\\ & \mu_{j}\geq 0 & \forall \;j\\ & s_{i}^{-},\;s_{r}^{+}\geq \;\text{0,} & \forall \;i,r \end{array}$$ (7)

#### Definition 3

DMU_o_, the DMU under test, is efficient if and only if an optimum is attained with all slacks zero in Model (7).

### 2.4. The fuzzy additive model

A hypothesis underlying DEA is that all the data take the form of specific numerical values. However, in a great variety of applications, the presence of imprecise or vague data like fuzzy data is undeniable. To this end, we present the additive Model (8) with fuzzy data in which ${\tilde{\text{x}}}_{ij}=(x^{\left(1\right)},x^{\left(2\right)},x^{\left(3\right)},x^{\left(4\right)})$ with the condition of x^(1)^ < x^(2)^ <x^(3)^ <x^(4)^ and ${\tilde{\text{y}}}_{rj}=(y^{\left(1\right)},y^{\left(2\right)},y^{\left(3\right)},y^{\left(4\right)})$ with the condition of y^(1)^ < y^(2)^ < y^(3)^ < y^(4)^ represent the *i*^*th*^ fuzzy input and *r*^*th*^ fuzzy output of DMU_*j*_, *j* = 1, 2,*…*, *n*, respectively under trapezoidal distribution.


$$\begin{array}{lll} Max & \sum_{i=1}^{m}s_{i}^{-}+\sum_{r=1}^{s}s_{r}^{+} & \\ s.t. & \sum_{j=1}^{n}\mu_{j}{\tilde{\text{x}}}_{\text{ij}}={\tilde{\text{x}}}_{\text{io}}-s_{i}^{-}\; & \forall \;i\\ & \sum_{j=1}^{n}\mu_{j}{\tilde{\text{y}}}_{\text{rj}}={\tilde{\text{y}}}_{\text{ro}}+s_{r}^{+} & \forall \;r\\ & \sum_{j=1}^{n}\mu_{j}=1 & \forall \;j\\ & \mu_{j}\geq 0\; & \forall \;j\\ & s_{i}^{-},\;s_{r}^{+}\geq \;\text{0,} & \forall \;i,r \end{array}$$ (8)

To tackle the uncertainty of fuzzy chance constraints in Model (8) and convert them to their equivalent crisp values by applying general fuzzy measure and chance constrained programming, Model (8) will be converted to the following model:

$$\begin{array}{lll} Max & \sum_{i=1}^{m}s_{i}^{-}+\sum_{r=1}^{s}s_{r}^{+} & \\ s.t.\; & GF\;\left\{\sum_{j=1}^{\text{n}}\mu_{j}{\tilde{\text{x}}}_{\text{ij}}\leq \;{\tilde{\text{x}}}_{\text{io}}-s_{i}^{-}\right\}\geq \gamma & \forall \;i\\ & GF\;\left\{\sum_{j=1}^{\text{n}}\mu_{j}{\tilde{\text{y}}}_{\text{rj}}\geq {\tilde{\text{y}}}_{\text{ro}}+s_{r}^{+}\;\right\}\geq \gamma & \forall \;r\\ & \sum_{j=1}^{n}\mu_{j}=1 & \forall \;j\\ & \mu_{j}\geq 0 & \forall \;j\\ & s_{i}^{-},\;s_{r}^{+}\geq \;\text{0,} & \forall \;i,r \end{array}$$ (9)

#### Definition 4

For the Model (9), DMU_o_ is an *γ*-efficient if an optimum is obtained with all slacks zero in Model (9) namely, the values of $s_{i}^{-*},\;(i=1,\;\ldots ,\;m)$ and $s_{r}^{+*},\;(r=1,\;\ldots ,\;s)$ are equal to zero where $s_{i}^{-*}$ and $s_{r}^{+*}$ are the optimal solutions of the Model (9).

From the properties of the *GF* approach and using relations (4) and (5), we can rewrite Model (9) as Model (10). It is significant that in the *GF* approach, an equivalent crisp of fuzzy chance constraints for the *γ* greater than or less than *η*, is not similar. So it has been applied a binary variable Ω and a large enough number *K* in Model (10) to the linearization of the incompatible constraints.


$$\begin{array}{l} Max\;\;\sum_{i=1}^{m}s_{i}^{-}+\sum_{r=1}^{s}s_{r}^{+}\\ s.t.\\ \sum_{\begin{array}{l} j=1\\ j\neq o \end{array}}^{n}\mu_{j}((\frac{\eta -\gamma }{\eta })x_{ij}^{\left(1\right)}+(\frac{\gamma }{\eta })x_{ij}^{\left(2\right)})+\mu_{o}((\frac{\gamma }{\eta })x_{io}^{\left(3\right)}+(\frac{\eta -\gamma }{\eta })x_{io}^{\left(4\right)})\leq ((\frac{\gamma }{\eta })x_{io}^{\left(3\right)}+(\frac{\eta -\gamma }{\eta })x_{io}^{\left(4\right)})-s_{i}^{-}+Κ\Omega ,\;\;\;\;\;\;\;\;\;\;\;\;\;\;\;\;\;\;\;\forall \;i\;\\ \sum_{\begin{array}{l} j=1\\ j\neq o \end{array}}^{n}\mu_{j}((\frac{1-\gamma }{1-\eta })x_{ij}^{\left(3\right)}+(\frac{\gamma -\eta }{1-\eta })x_{ij}^{\left(4\right)})+\mu_{o}((\frac{\gamma -\eta }{1-\eta })x_{io}^{\left(1\right)}+(\frac{1-\gamma }{1-\eta })x_{io}^{\left(2\right)})\leq ((\frac{\gamma -\eta }{1-\eta })x_{io}^{\left(1\right)}+(\frac{1-\gamma }{1-\eta })x_{io}^{\left(2\right)})-s_{i}^{-}+Κ(1-\Omega ),\;\;\forall \;i\;\\ \sum_{\begin{array}{l} j=1\\ j\neq o \end{array}}^{n}\mu_{j}((\frac{\gamma }{\eta })y_{rj}^{\left(3\right)}+(\frac{\eta -\gamma }{\eta })y_{rj}^{\left(4\right)})+\mu_{o}((\frac{\eta -\gamma }{\eta })y_{ro}^{\left(1\right)}+(\frac{\gamma }{\eta })y_{ro}^{\left(2\right)})\geq ((\frac{\eta -\gamma }{\eta })y_{ro}^{\left(1\right)}+(\frac{\gamma }{\eta })y_{ro}^{\left(2\right)})+s_{r}^{+}-Κ\Omega ,\;\;\;\;\;\;\;\;\;\;\;\;\;\;\;\;\;\forall \;r\\ \sum_{\begin{array}{l} j=1\\ j\neq o \end{array}}^{n}\mu_{j}((\frac{\gamma -\eta }{1-\eta })y_{rj}^{\left(1\right)}+(\frac{1-\gamma }{1-\eta })y_{rj}^{\left(2\right)})+\mu_{o}((\frac{1-\gamma }{1-\eta })y_{ro}^{\left(3\right)}+(\frac{\gamma -\eta }{1-\eta })y_{ro}^{\left(4\right)})\geq ((\frac{1-\gamma }{1-\eta })y_{ro}^{\left(3\right)}+(\frac{\gamma -\eta }{1-\eta })y_{ro}^{\left(4\right)})+s_{r}^{+}-Κ(1-\Omega ),\;\forall \;r\;\;\;\;\;\;\;\;\;\\ \sum_{j=1}^{n}\mu_{j}=1\;\;\;\;\;\;\;\;\;\;\;\;\;\;\;\;\;\;\;\;\;\;\forall \;j\\ \;\;\;\;\;\;\gamma \leq \eta \text{+}Κ\Omega \\ \;\;\;\;\;\;\gamma >\eta -Κ(1-\Omega )\\ \;\;\;\;\;\Omega \in \{0,1\}\;\;\;\;\;\;\\ \;\;\;\;\;\mu_{j}\geq 0\;\;\;\;\;\;\;\;\;\;\;\;\;\;\;\;\;\;\;\;\;\;\;\;\;\;\forall \;j\;\\ \;\;\;\;\;s_{i}^{-},\;s_{r}^{+}\geq \;0,\;\;\;\;\;\;\;\;\;\;\;\;\;\;\;\;\;\;\;\forall \;i,r\;\;\;\\ \;\;\; \end{array}$$ (10)

#### Theorem 2.1

If DMU_o_ is an *γ*-inefficient DMU, then an optimum is obtained with $\mu_{o}^{*}=0$ in Model (9).

*Proof* Let us hypothesize that for a fixed *γ*, the solution $\left(\mu_{j}^{*},\mu_{o}^{*},s_{i}^{-*},s_{r}^{+*}\right)$ is the optimal value of Model (9) with the optimal objective $\sum_{i=1}^{m}s_{i}^{-*}+\sum_{r=1}^{s}s_{r}^{+*}$, the theorem is proved if $\mu_{o}^{*}=0$. Otherwise, since DMU_o_ is inefficient, there is at least one $s_{i}^{-*}>0\;(i=1,\ldots ,m)$ or $s_{r}^{+*}>0\;(r=1,\ldots ,s)$ such that $\mu_{o}^{*}>0$. Suppose that $s_{k}^{-*}>0\;(k\in \left\{1,\ldots ,m\right\})$. If $\mu_{o}^{*}=1$, in that case $GF\{{\tilde{x}}_{ok}\leq {\tilde{x}}_{ok}-s_{k}^{-*}\}=0$. This antilogy states that $\mu_{o}^{*}\neq 1$.

Now, we consider the case $0<\mu_{o}^{*}<1$.


$$\begin{array}{l} GF\;\left\{\sum_{j=1}^{\text{n}}\mu_{j}^{*}{\tilde{x}}_{\text{ij}}\leq \;{\tilde{x}}_{\text{io}}-s_{i}^{-*}\right\}=GF\;\left\{\sum_{j=1,j\neq o}^{\text{n}}\mu_{j}^{*}{\tilde{x}}_{\text{ij}}+\mu_{o}^{*}{\tilde{x}}_{\text{io}}\leq \;{\tilde{x}}_{\text{io}}-s_{i}^{-*}\right\}\\ \;\;\;\;\;\;\;\;\;\;\;\;\;\;\;\;\;\;\;\;\;\;\;\;\;\;\;\;\;\;\;\;\;\;\;\;\;\;\;\;\;\;=GF\;\left\{\sum_{j=1,j\neq o}^{\text{n}}\mu_{j}^{*}{\tilde{x}}_{\text{ij}}\leq \;(1-\mu_{o}^{*}){\tilde{x}}_{\text{io}}-s_{i}^{-*}\right\}\\ \;\;\;\;\;\;\;\;\;\;\;\;\;\;\;\;\;\;\;\;\;\;\;\;\;\;\;\;\;\;\;\;\;\;\;\;\;\;\;\;\;\;=GF\;\left\{\frac{\sum_{j=1,j\neq o}^{\text{n}}\mu_{j}^{*}{\tilde{x}}_{\text{ij}}}{\left(1-\mu_{o}^{*}\right)}\leq \;{\tilde{x}}_{\text{io}}-\frac{s_{i}^{-*}}{\left(1-\mu_{o}^{*}\right)}\right\}\geq \gamma ,\;\;i=1,\ldots ,m.\\ \; \end{array}$$

In a similar way,

$$GF\;\left\{\sum_{j=1}^{\text{n}}\mu_{j}^{*}{\tilde{\text{y}}}_{\text{rj}}\geq {\tilde{\text{y}}}_{\text{ro}}+s_{r}^{+*}\right\}=GF\;\left\{\frac{\sum_{j=1,j\neq o}^{\text{n}}\mu_{j}^{*}{\tilde{\text{y}}}_{\text{rj}}}{\left(1-\mu_{o}^{*}\right)}\geq \;{\tilde{\text{y}}}_{\text{ro}}+\frac{s_{r}^{+*}}{\left(1-\mu_{o}^{*}\right)}\right\}\geq \gamma ,\;\;r=1,\ldots ,s.\;$$

It is straightforward $\frac{\sum_{j=1,j\neq o}^{\text{n}}\mu_{j}^{*}{\tilde{\text{y}}}_{\text{rj}}}{\left(1-\mu_{o}^{*}\right)}=1$. Hence $\left(\frac{\mu_{1}^{*}}{1-\mu_{o}^{*}},\;\ldots ,\;\frac{\mu_{o-1}^{*}}{1-\mu_{o}^{*}},\;0,\;\;\frac{\mu_{o+1}^{*}}{1-\mu_{o}^{*}},\;\ldots ,\;\frac{\mu_{n}^{*}}{1-\mu_{o}^{*}}\right)$ is a feasible solution for Model (9) such that the objective value for this solution is $\frac{1}{1-\mu_{o}^{*}}\left(\sum_{i=1}^{m}s_{i}^{-*}+\sum_{r=1}^{s}s_{r}^{+*}\right)$. And since $0<\mu_{o}^{*}<1$, it contradicts the assumption $\left(\sum_{i=1}^{m}s_{i}^{-*}+\sum_{r=1}^{s}s_{r}^{+*}\right)$. Therefore $\mu_{o}^{*}=0$.

## 3. Sensitivity analysis with fuzzy data

### 3.1. Stability radius for efficient DMUs

Suppose that DMU_o_ is an *γ*-efficient DMU. The aim is to find scalars *ρ*_*i*_ (*i* = 1,…, *m*) and *φ*_*r*_ (*r* = 1,…,*s*) such that if *i*^*th*^ input of DMU_o_ is increased by *ρ*_*i*_ and *r*^*th*^ output of DMU_o_ is decreased by *φ*_*r*_, then DMU_o_ remains *γ*-efficient. For this purpose, we consider the following model:

$$\begin{array}{lll} Min & \sum_{i=1}^{m}\rho_{i}+\sum_{r=1}^{s}\varphi_{r} & \\ s.t. & GF\;\left\{\sum_{\begin{array}{l} j=1\\ j\neq 0 \end{array}}^{\text{n}}\mu_{j}{\tilde{\text{x}}}_{\text{ij}}\leq \;{\tilde{\text{x}}}_{\text{io}}+\rho_{i}\right\}\geq \gamma & \forall \;i\\ & GF\;\left\{\sum_{\begin{array}{l} j=1\\ j\neq o \end{array}}^{\text{n}}\mu_{j}{\tilde{\text{y}}}_{\text{rj}}\geq {\tilde{\text{y}}}_{\text{ro}}-\varphi_{r}\;\right\}\geq \gamma & \forall \;r\\ & \sum_{\begin{array}{l} j=1\\ j\neq o \end{array}}^{n}\mu_{j}=1 & \forall \;j\\ & \mu_{j}\geq 0 & \forall \;j\\ & \rho_{i},\;\varphi_{r}\geq \;\text{0,} & \forall \;i,r \end{array}$$ (11)

#### Theorem 3.1

Theorize that DMU_o_ is an *γ*-efficient DMU, and $\left(\mu_{1}^{*},\ldots ,\mu_{n}^{*},\rho_{1}^{*},\ldots ,\rho_{m}^{*},\varphi_{1}^{*},\ldots ,\varphi_{s}^{*}\right)$ is an optimal solution of Model (11). In this case $\tilde{DMU_{o}}$ with inputs $\text{(}{\tilde{\text{x}}}_{\text{io}}+\rho_{i}^{*}\text{),}\;(i=1,\ldots ,m)$ and outputs $\text{(}{\tilde{\text{y}}}_{\text{ro}}-\varphi_{r}^{*}\text{),}\;(r=1,\ldots ,s)$ remains an *γ*-efficient DMU.

*Proof* Assume that $\left({\overset{⌢}{\mu }}_{j},{\overset{⌢}{\mu }}_{o},s_{i}^{-*},s_{r}^{+*}\right)$ is the optimal value of Model (9) when evaluating $\tilde{DMU_{o}}$ therefore we have:

$$\begin{array}{lll} GF & \left\{\sum_{j=1,j\neq o}^{\text{n}}{\overset{⌢}{\mu }}_{j}{\tilde{\text{x}}}_{\text{ij}}+{\overset{⌢}{\mu }}_{o}\text{(}{\tilde{\text{x}}}_{\text{io}}+\rho_{i}^{*}\text{)}\leq \;\text{(}{\tilde{\text{x}}}_{\text{io}}+\rho_{i}^{*}\text{)}-s_{i}^{-*}\right\}\geq \gamma & \forall \;i\\ GF & \left\{\sum_{j=1,j\neq o}^{\text{n}}{\overset{⌢}{\mu }}_{j}{\tilde{\text{y}}}_{\text{rj}}+{\overset{⌢}{\mu }}_{o}\text{(}{\tilde{\text{y}}}_{\text{ro}}-\varphi_{r}^{*}\text{)}\geq \text{(}{\tilde{\text{y}}}_{\text{ro}}-\varphi_{r}^{*}\text{)}+s_{r}^{+*}\;\right\}\geq \gamma & \forall \;r \end{array}$$

Assume that $\tilde{DMU_{o}}$ is inefficient thus from Theorem 2.1, ${\overset{⌢}{\mu }}_{o}=0$ and we have:

$$\begin{array}{lll} GF & \left\{\sum_{j=1,j\neq o}^{\text{n}}{\overset{⌢}{\mu }}_{j}{\tilde{\text{x}}}_{\text{ij}}\leq \;\text{(}{\tilde{\text{x}}}_{\text{io}}+\rho_{i}^{*}\text{)}-s_{i}^{-*}\right\}\geq \gamma & \forall \;i\\ GF & \left\{\sum_{j=1,j\neq o}^{\text{n}}{\overset{⌢}{\mu }}_{j}{\tilde{\text{y}}}_{\text{rj}}\geq \text{(}{\tilde{\text{y}}}_{\text{ro}}-\varphi_{r}^{*}\text{)}+s_{r}^{+*}\;\right\}\geq \gamma & \forall \;r \end{array}$$

We set ${\hat{\rho }}_{i}=\rho_{i}^{*}-s_{i}^{-*}$ and ${\hat{\varphi }}_{r}=\varphi_{r}^{*}-s_{r}^{+*}$. Thus $\left({\overset{⌢}{\mu }}_{1},\ldots ,{\overset{⌢}{\mu }}_{n},{\hat{\rho }}_{1},\ldots ,{\hat{\rho }}_{m},{\hat{\varphi }}_{1},\ldots ,{\hat{\varphi }}_{s}\right)$ is a feasible solution for Model (11) and the objective value is $\sum_{i=1}^{m}{\hat{\rho }}_{i}+\sum_{r=1}^{s}{\hat{\varphi }}_{r}<\sum_{i=1}^{m}\rho_{i}^{*}+\sum_{r=1}^{s}\varphi_{r}^{*}$, which leads to a contradiction with the assumption.

From the above analysis, if DMU_o_ is an *γ*-efficient DMU, and $\left(\mu_{1}^{*},\ldots ,\mu_{n}^{*},\rho_{1}^{*},\ldots ,\rho_{m}^{*},\varphi_{1}^{*},\ldots ,\varphi_{s}^{*}\right)$ is an optimal solution of Model (11), then for each *ρ*_*i*_ (*i* = 1,…, *m*) and *φ*_*r*_ (*r* = 1,…,*s*), where $\rho_{i}\in [0,\rho_{i}^{*}]$, (*i* = 1, …,*m*) and $\varphi_{r}\in [0,\varphi_{r}^{*}]$, (*r* = 1, …,*m*) if $\text{(}{\tilde{\text{x}}}_{\text{io}}+\rho_{i})$, (*i* = 1, …,*m*) and $\text{(}{\tilde{\text{y}}}_{\text{ro}}-\varphi_{r})$, (*r* = 1, …,*m*) then DMU_o_ remains *γ*-efficient.

We can rewrite Model (11) as a fuzzy DEA crisp model for the *γ* greater than or less than η according to the *GF* measure as follows:

$$\begin{array}{l} Min\;\;\sum_{i=1}^{m}\rho_{i}+\sum_{r=1}^{s}\varphi_{r}\\ s.t.\\ \sum_{\begin{array}{l} j=1\\ j\neq o \end{array}}^{n}\mu_{j}((\frac{\eta -\gamma }{\eta })x_{ij}^{\left(1\right)}+(\frac{\gamma }{\eta })x_{ij}^{\left(2\right)})\leq ((\frac{\gamma }{\eta })x_{io}^{\left(3\right)}+(\frac{\eta -\gamma }{\eta })x_{io}^{\left(4\right)})+\rho_{i}+Κ\Omega ,\;\;\;\;\;\;\;\;\;\;\;\;\;\;\;\;\;\;\;\;\;\forall \;i\;\\ \sum_{\begin{array}{l} j=1\\ j\neq o \end{array}}^{n}\mu_{j}((\frac{1-\gamma }{1-\eta })x_{ij}^{\left(3\right)}+(\frac{\gamma -\eta }{1-\eta })x_{ij}^{\left(4\right)})\leq ((\frac{\gamma -\eta }{1-\eta })x_{io}^{\left(1\right)}+(\frac{1-\gamma }{1-\eta })x_{io}^{\left(2\right)})+\rho_{i}+Κ(1-\Omega ),\;\;\;\forall \;i\;\\ \sum_{\begin{array}{l} j=1\\ j\neq o \end{array}}^{n}\mu_{j}((\frac{\gamma }{\eta })y_{rj}^{\left(3\right)}+(\frac{\eta -\gamma }{\eta })y_{rj}^{\left(4\right)})\geq ((\frac{\eta -\gamma }{\eta })y_{ro}^{\left(1\right)}+(\frac{\gamma }{\eta })y_{ro}^{\left(2\right)})-\varphi_{r}-Κ\Omega ,\;\;\;\;\;\;\;\;\;\;\;\;\;\;\;\;\;\;\;\;\forall \;r\\ \sum_{\begin{array}{l} j=1\\ j\neq o \end{array}}^{n}\mu_{j}((\frac{\gamma -\eta }{1-\eta })y_{rj}^{\left(1\right)}+(\frac{1-\gamma }{1-\eta })y_{rj}^{\left(2\right)})\geq ((\frac{1-\gamma }{1-\eta })y_{ro}^{\left(3\right)}+(\frac{\gamma -\eta }{1-\eta })y_{ro}^{\left(4\right)})-\varphi_{r}-Κ(1-\Omega ),\;\;\forall \;r\;\;\;\;\;\;\;\;\;\\ \sum_{\begin{array}{l} j=1\\ j\neq o \end{array}}^{n}\mu_{j}=1\;\;\;\;\;\;\;\;\;\;\;\;\;\;\;\;\;\;\;\;\;\;\forall \;j\\ \;\;\;\;\;\;\gamma \leq \eta +\text{K}\Omega \\ \;\;\;\;\;\;\gamma >\eta -\text{K}(1-\Omega )\\ \;\;\;\;\;\Omega \in \{0,1\}\;\;\;\;\;\;\\ \;\;\;\;\;\mu_{j}\geq 0\;\;\;\;\;\;\;\;\;\;\;\;\;\;\;\;\;\;\;\;\;\;\;\;\;\;\forall \;j\;\\ \;\;\;\;\;\rho_{i},\;s_{r}^{+}\geq \;0,\;\;\;\;\;\;\;\;\;\;\;\;\;\;\;\;\;\;\;\forall \;i,r\;\;\;\\ \;\;\; \end{array}$$ (12)

### 3.2. Stability radius for inefficient DMUs

In this case, we imagine that DMU_o_ is an *γ*-inefficient DMU; that is, $\mu_{o}^{*}=0$. The aim is to find scalars *ρ*_*i*_ (*i* = 1,…, *m*) and *φ*_*r*_ (*r* = 1,…,*s*) such that if *i*^*th*^ input of DMU_o_ is decreased by *ρ*_*i*_ and *r*^*th*^ output of DMU_o_ is increased by *φ*_*r*_, then DMU_o_ remains *γ*-inefficient. To this end, we have the following model:

$$\begin{array}{lll} Max & \sum_{i=1}^{m}\rho_{i}+\sum_{r=1}^{s}\varphi_{r} & \\ s.t. & GF\;\left\{\sum_{j=1}^{\text{n}}\mu_{j}{\tilde{\text{x}}}_{\text{ij}}\leq \;{\tilde{\text{x}}}_{\text{io}}-\rho_{i}\right\}\geq \gamma & \forall \;i\\ & GF\;\left\{\sum_{j=1}^{\text{n}}\mu_{j}{\tilde{\text{y}}}_{\text{rj}}\geq {\tilde{\text{y}}}_{\text{ro}}+\varphi_{r}\;\right\}\geq \gamma & \forall \;r\\ & \sum_{j=1}^{n}\mu_{j}=1 & \forall \;j\\ & \mu_{j}\geq 0 & \forall \;j\\ & \rho_{i},\;\varphi_{r}\geq \;\text{0,} & \forall \;i,r \end{array}$$ (13)

#### Theorem 3.2

Imagine that DMU_o_ is an *γ*-inefficient DMU, and $\left(\mu_{1}^{*},\ldots ,\mu_{n}^{*},\rho_{1}^{*},\ldots ,\rho_{m}^{*},\varphi_{1}^{*},\ldots ,\varphi_{s}^{*}\right)$ is the optimal solution of Model (13). In this case $\tilde{DMU_{o}}$ with inputs $\text{(}{\tilde{\text{x}}}_{\text{io}}-\rho_{i}\text{),}\;(i=1,\ldots ,m)$ where $\rho_{i}\in [0,\rho_{i}^{*}),\;(i=1,\ldots ,m)$ and outputs $\text{(}{\tilde{\text{y}}}_{\text{ro}}+\varphi_{r}\text{),}\;(r=1,\ldots ,s)$ where $\varphi_{r}\in [0,\varphi_{r}^{*}),\;(r=1,\ldots ,s)$ remains an *γ*-inefficient DMU, i.e. $\mu_{o}^{*}=0$.

*Proof*. The theorem’s proof is like to that of Theorem 2.1 and is eliminated.

## 4. Numerical example

### 4.1. Illustrative example

This section provides an example with the data defined in Table 1 to demonstrate the stability analysis. The numerical example is related to five DMUs with two fuzzy inputs and two fuzzy outputs in the form of a trapezoidal fuzzy number.

**Table 1:** Data set of five DMUs with two fuzzy inputs and two fuzzy outputs.

DMUs	DMU_A_	DMU_B_	DMU_C_	DMU_D_	DMU_E_
Input(I_1_)	(0.5, 1, 1.5, 2)	(2.25, 2.75, 3.25, 3.75)	(3.25, 3.75, 4.25, 4.75)	(2, 4, 6, 8)	(3.5, 4.5, 5.5, 6.5)
Input(I_2_)	(1.5, 1.75, 2, 2.25)	(2, 3, 4, 5)	(2.25, 2.5, 2.75, 3)	(4, 4.5, 5, 5.5)	(4.5, 5, 5.5, 6)
Output(O_1_)	(3, 4, 5, 6)	(1, 3, 5, 7)	(1, 2, 3, 4)	(0.5, 1, 1.5, 2)	(1.5, 2, 2.5, 3)
Output(O_2_)	(2, 2.75, 3.5, 4.25)	(1.5, 2.5, 3.5, 4.5)	(4, 4.5, 5, 5.5)	(2, 3, 4, 5)	(0.25, 0.5, 0.75, 1)

We evaluated DMUs using Model (9) in the case of *γ* < *η* with confidence level *γ* = 0.2 and adjustment parameter *η* = 0.4, the results presented in Table 2.

**Table 2:** Results of evaluating DMUs in the case of *γ* < *η* by Model (9).

DMU_j_	$\sum_{i=1}^{m}s_{i}^{-*}+\sum_{r=1}^{s}s_{r}^{+*}$	$\left(\mu_{A}^{*},\mu_{B}^{*},\mu_{C}^{*},\mu_{D}^{*},\mu_{E}^{*}\right)$	Efficiency
DMU_A_	0	(1,0,0,0,0)	Efficient
DMU_B_	11	(1,0,0,0,0)	Inefficient
DMU_C_	0	(0,0,1,0,0)	Efficient
DMU_D_	16	(1,0,0,0,0)	Inefficient
DMU_E_	16.625	(1,0,0,0,0)	Inefficient

Using Model (9), DMU_A_ and DMU_C_ are efficient, while DMU_B_, DMU_D_, and DMU_E_ are inefficient. Sensitivity analysis for efficient DMUs using Model (11) is shown in Table 3.

**Table 3:** Sensitivity analysis for efficient DMUs in the case of *γ* < *η* by Model (11).

DMU_j_	$\rho_{1}^{*}$	$\rho_{2}^{*}$	$\varphi_{1}^{*}$	$\varphi_{2}^{*}$
DMU_A_	0.75	0.375	0	0
DMU_C_	0	0	0	0.077

To interpret Table 3, we say that the second and third columns show the maximum increase in the first and second input of efficient DMUs, respectively, while maintaining their efficiency. Similarly, the fourth and fifth columns show the maximum decrease in the first and second output of efficient DMUs while keeping the efficiency of DMUs. For instance, consider the first row of Table 3, DMU_A_ = (*x*_*1A*_, *x*_*2A*_, *y*_*1A*_, *y*_*2A*_) remains efficient if $\tilde{DMU_{A}}=\left(x_{1A}+{\overset{⌢}{\rho }}_{1},x_{2A}+{\overset{⌢}{\rho }}_{2},y_{1A},y_{2A}\right)$ in which $0\leq {\overset{⌢}{\rho }}_{1}\leq 0.75$ and $0\leq {\overset{⌢}{\rho }}_{2}\leq 0.375$.

Also, Table 4 gives sensitivity analysis for inefficient DMUs which estimated by Model (13).

**Table 4:** Sensitivity analysis for inefficient DMUs in the case of *γ* < *η* by Model (13).

DMU_j_	$\rho_{1}^{*}$	$\rho_{2}^{*}$	$\varphi_{1}^{*}$	$\varphi_{2}^{*}$
DMU_B_	2.75	2.875	3.5	1.875
DMU_D_	6.25	3.625	4.75	1.375
DMU_E_	5.25	4.125	3.75	3.5

The second and third columns document the lower bounds of variation ranges in the first and second input of inefficient DMUs, respectively, while maintaining their inefficiency. Similarly, the fourth and fifth columns document the upper bounds of variation ranges in the first and second output of inefficient DMUs while keeping the inefficiency of DMUs. For instance, we consider the first row of Table 4, DMU_B_ = (*x*_*1B*_, *x*_*2B*_, *y*_*1B*_, *y*_*2B*_) remains inefficient if $\tilde{DMU_{B}}=\left(x_{1B}-{\overset{⌢}{\rho }}_{1},x_{2B}-{\overset{⌢}{\rho }}_{2},y_{1B}+{\overset{⌢}{\varphi }}_{1},y_{2B}+{\overset{⌢}{\varphi }}_{2}\right)$ in which $0\leq {\overset{⌢}{\rho }}_{1}\leq 2.75$, $0\leq {\overset{⌢}{\rho }}_{2}\leq 2.875$, $0\leq {\overset{⌢}{\varphi }}_{1}\leq 3.5$ and $0\leq {\overset{⌢}{\varphi }}_{2}\leq 1.875$.

Similar to the above discussion will be presented in the case of *γ* < *η* with confidence level 0.8 and adjustment parameter 0.2. The results of the evaluating DMUs are shown in Table 5.

**Table 5:** Results of evaluating DMUs in the case of *γ* > *η* by Model (9).

DMU_j_	$\sum_{i=1}^{m}s_{i}^{-*}+\sum_{r=1}^{s}s_{r}^{+*}$	$\left(\mu_{A}^{*},\mu_{B}^{*},\mu_{C}^{*},\mu_{D}^{*},\mu_{E}^{*}\right)$	Efficiency
DMU_A_	0	(1,0,0,0,0)	Efficient
DMU_B_	0	(0,1,0,0,0)	Efficient
DMU_C_	0	(0,0,1,0,0)	Efficient
DMU_D_	0	(0,0,0,1,0)	Efficient
DMU_E_	5.937	(1,0,0,0,0)	Inefficient

As shown in Table 5, in the case of *γ* < *η*, four DMUs, namely DMU_A_, DMU_B_, DMU_C_ and DMU_D_, are efficient, and DMU_E_ is inefficient. Sensitivity analysis for the efficient DMUs using Model (11) and sensitivity analysis for the inefficient DMU using Model (13) are shown in Tables 6 and 7, respectively.

**Table 6:** Sensitivity analysis for efficient DMUs in the case of *γ* > *η* by Model (11).

DMU_j_	$\rho_{1}^{*}$	$\rho_{2}^{*}$	$\varphi_{1}^{*}$	$\varphi_{2}^{*}$
DMU_A_	3.983	1.408	4.468	0
DMU_B_	0	0	3.25	2.063
DMU_C_	0	0	0.5	3.188
DMU_D_	0	0	0	2.122

**Table 7:** Sensitivity analysis for inefficient DMU in the case of *γ* > *η* by Model (13).

DMU_j_	$\rho_{1}^{*}$	$\rho_{2}^{*}$	$\varphi_{1}^{*}$	$\varphi_{2}^{*}$
DMU_E_	1.875	2.438	0.375	1.25

The interpretation of the last two tables is similar to Tables 4 and 5. Also, from the comparison between Tables 2 and 5, we find that in the case of *γ* < *η*, the number of efficient units is more than in the case of *γ* > *η*.

### 4.2. An applicable data set

A public system of compulsory insurance that should be provided by the government for all persons of society is called social security. Utilization of social security benefits, such as retirement, unemployment, old age, disability, loss of caretaker, helplessness, accidents, and injuries, requiring insurance or non-insurance medical and health care services, is a public right for all individuals in society. By considering all the things above, a comprehensive social security system is established to provide the aforementioned services.

In this section, considering the proposed method, the sensitivity analysis for efficient and inefficient DMUs is illustrated for evaluating 12 branches of the Social Security Organization in Tehran. Each branch (DMU) applies two inputs (total number of computers, and number of staff) to generate two outputs (sum of insured individuals’ contracts, and all individuals under insurance). Also, input and output data in the form of trapezoidal fuzzy numbers are documented in Tables 8 and 9, respectively.

**Table 8:** Inputs of 12branch of social security organization in Tehran.

DMU_j_	Total number of computers (I_1_)	Number of staff (I_2_)
DMU_1_	(84,84.16,90.16,91.99)	(106,107.25,110.25,112)
DMU_2_	(92,92.58,93.58,93.99)	(82,83,86.5,88)
DMU_3_	(92,92,92,92)	(85,86,88.5,90)
DMU_4_	(88,88.416,89.41,89.99)	(75,76.83,79.83,80.99)
DMU_5_	(99.99,100.08,100.58,100.99)	(82,83,85.5,87)
DMU_6_	(83,83,83,83)	(89,89.83,91.33,91.99)
DMU_7_	(91,91.33,91.83,91.99)	(94,97.91,104.91,107.99)
DMU_8_	(90,90.5,91.5,92)	(96,96.66,98.66,99.99)
DMU_9_	(93,93.75,95.25,96)	(91,91.66,93.16,93.99)
DMU_10_	(107,107.33,109.33,110.99)	(102,102,102,102)
DMU_11_	(85,86,88,89)	(77,77.75,79.25,80)
DMU_12_	(78,78,78,78)	(94,95.58,99.08,100.99)

**Table 9:** Outputs of 12 branch of social security organization in Tehran.

DMU_j_	Sum of insured individuals’ contracts (O_1_)	All individuals under insurance (O_2_)
DMU_1_	(43,50.58,77.58,96.99)	(85399,85810.25,86720.75,87220)
DMU_2_	(28,31.83,39.33,42.99)	(87716,88307.25,89574.25,90250)
DMU_3_	(11,14.16,22.16,26.99)	(42900,43963.83,46148.83,47269.99)
DMU_4_	(0,7.83,18.83,21.99)	(36740,36770.25,36826.25,36852)
DMU_5_	(29,56.14,204.41,324.99)	(27978,28475.91,30958.41,32942.99)
DMU_6_	(9,18.08,35.08,42.99)	(52127,53398,54343,54817)
DMU_7_	(13,16.83,22.83,24.99)	(78550,80892.75,86173.25,89111)
DMU_8_	(47,70.08,111.08,128.99)	(32585,34220.66,36649.66,37442.99)
DMU_9_	(10,20.16,42.66,54.99)	(35469,35577.08,35851.08,36016.99)
DMU_10_	(30,36,49.5,57)	(56144,56814.41,58150.41,58815.99)
DMU_11_	(11,14.33,22.33,26.99)	(38004,38225.08,38614.58,38782.99)
DMU_12_	(81,129.75,210.25,242)	(36652,38567.16,42390.16,44297.99)

By considering the adjustment parameter of 0.75, we evaluated the performance of these 12 branches of the Social Security Organization for some confidence levels in the case of *γ* ≤ 0.75. The evaluation results are documented in Table 10.

**Table 10:** Evaluation of 12 DMUs in the case of *γ* ≤ 0.75.

DMU_j_	Confidence levels (*γ* ≤ 0.75)							
	*γ* = 0		*γ* = 0.25		*γ* = 0.5		*γ* = 0.75	
	Z*	Efficiency	Z*	Efficiency	Z*	Efficiency	Z*	Efficiency
DMU_1_	4856.915	Inefficient	2223.679	Inefficient	0	Efficient	0	Efficient
DMU_2_	0	Efficient	0	Efficient	0	Efficient	0	Efficient
DMU_3_	47389.990	Inefficient	46737.041	Inefficient	46088.957	Inefficient	45445.495	Inefficient
DMU_4_	39877.121	Inefficient	35507.664	Inefficient	28822.224	Inefficient	22056.104	Inefficient
DMU_5_	62299.980	Inefficient	61897.330	Inefficient	60941.641	Inefficient	56921.478	Inefficient
DMU_6_	11819.975	Inefficient	9717.656	Inefficient	7577.350	Inefficient	5398.696	Inefficient
DMU_7_	11752.230	Inefficient	10653.049	Inefficient	9560.071	Inefficient	8472.985	Inefficient
DMU_8_	57456.796	Inefficient	56119.518	Inefficient	54669.833	Inefficient	51685.650	Inefficient
DMU_9_	54829.980	Inefficient	54563.043	Inefficient	54296.107	Inefficient	54029.170	Inefficient
DMU_10_	34157.980	Inefficient	33704.960	Inefficient	33251.940	Inefficient	32798.920	Inefficient
DMU_11_	24787.545	Inefficient	18499.863	Inefficient	12096.933	Inefficient	5778.021	Inefficient
DMU_12_	0	Efficient	0	Efficient	0	Efficient	0	Efficient

Table 10 represents the amount of objective function of Model (10) for all DMUs to determine the efficiency classification of DMUs, in which $Z^{*}=\sum_{i=1}^{m}s_{i}^{-*}+\sum_{r=1}^{s}s_{r}^{+*}$. As shown in Table 10, DMU_2_and DMU_12_are efficient for all confidence levels less than or equal to 0.75, while DMU_1_is efficient only for the confidence levels of 0.5 and 0.75, and the other DMUs are inefficient. Table 11 reports the stability radius for efficient DMUs with various confidence levels of *γ* ≤ 0.75.

**Table 11:** Sensitivity analysis for efficient DMUs in the case of *γ* ≤ 0.75.

DMU_j_	Confidence level (*γ* = 0)			
	$\rho_{1}^{*}$	$\rho_{2}^{*}$	$\varphi_{1}^{*}$	$\varphi_{2}^{*}$
DMU_2_	0	5.579	0	0
DMU_12_	5.704	0	0	0
DMU_j_	Confidence level (*γ* = 0.25)			
	$\rho_{1}^{*}$	$\rho_{2}^{*}$	$\varphi_{1}^{*}$	$\varphi_{2}^{*}$
DMU_2_	0	8.493	0	0
DMU_12_	7.294	0	0	0
DMU_j_	Confidence level (*γ* = 0.5)			
	$\rho_{1}^{*}$	$\rho_{2}^{*}$	$\varphi_{1}^{*}$	$\varphi_{2}^{*}$
DMU_1_	0.239	0	0	0
DMU_2_	0	9.607	7.003	957.667
DMU_12_	9.418	0	0	0
DMU_j_	Confidence level (*γ* = 0.75)			
	$\rho_{1}^{*}$	$\rho_{2}^{*}$	$\varphi_{1}^{*}$	$\varphi_{2}^{*}$
DMU_1_	1.240	0	0	0
DMU_2_	0	20.750	0	1586.500
DMU_12_	12.709	0	0	0

The second and third columns report the upper bounds of variation ranges in the total number of computers and the number of staff for efficient branches, respectively, while maintaining their efficiency. Similarly, the fourth and fifth columns report the lower bounds of variation ranges in the sum of insured individuals’ contracts and all individuals under insurance for efficient branches while keeping their efficiency. For instance, consider DMU_2_ with the confidence level of *γ* = 0.5 in Table 11. DMU_2_ = (*x*_*12*_, *x*_*22*_, *y*_*12*_, *y*_*22*_) remains efficient if $\tilde{DMU_{2}}=\left(x_{12},\;x_{22}+{\overset{⌢}{\rho }}_{2},\;y_{12}-{\overset{⌢}{\varphi }}_{1},\;y_{22}-{\overset{⌢}{\varphi }}_{2}\right)$ in which, $0\leq {\overset{⌢}{\rho }}_{2}\leq 9.607$, $0\leq {\overset{⌢}{\varphi }}_{1}\leq 7.003$ and $0\leq {\overset{⌢}{\varphi }}_{2}\leq 957.667$. In other words, if the number of computers in Branch 2 remains fixed and the number of staff increases by a maximum of 9.607, also if the sum of insured individuals’ contracts decreases by a maximum of 7.003 and all individuals under insurance decrease by a maximum of 957.667, then Branch 2 will remain efficient.

In the following, Table 12 reports the stability radius for inefficient DMUs with various confidence levels of *γ* ≤ 0.75.

**Table 12:** Sensitivity analysis for inefficient DMUs in the case of *γ* ≤ 0.75.

DMU_j_	Confidence level (*γ* = 0)			
	$\rho_{1}^{*}$	$\rho_{2}^{*}$	$\varphi_{1}^{*}$	$\varphi_{2}^{*}$
DMU_1_	0	29.946	0	4826.969
DMU_3_	0	8	31.990	47350
DMU_4_	0	0	42.866	39834.255
DMU_5_	8.990	5	13.990	62272
DMU_6_	0	0	153.997	11665.978
DMU_7_	0	25.960	30.058	11696.212
DMU_8_	0.594	16.208	0	57439.994
DMU_9_	4	11.990	32.990	54781
DMU_10_	18.990	20	12.990	34106
DMU_11_	0	0	40.505	24747.040
DMU_j_	Confidence level (*γ* = 0.25)			
	$\rho_{1}^{*}$	$\rho_{2}^{*}$	$\varphi_{1}^{*}$	$\varphi_{2}^{*}$
DMU_1_	0	25.707	0	2197.972
DMU_3_	0	6.595	30.875	46699.572
DMU_4_	0	0	33.906	35473.758
DMU_5_	8.660	4.167	3.723	61880.780
DMU_6_	0	0	147.178	9570.478
DMU_7_	0	23.871	29.030	10600.148
DMU_8_	1.798	10.829	0	56106.891
DMU_9_	3.557	11.380	28.383	54519.723
DMU_10_	18.243	19.667	9.770	33657.280
DMU_11_	0	0	35.531	18464.332
DMU_j_	Confidence level (*γ* = 0.5)			
	$\rho_{1}^{*}$	$\rho_{2}^{*}$	$\varphi_{1}^{*}$	$\varphi_{2}^{*}$
DMU_3_	0	5.205	29.475	46054.277
DMU_4_	0.403	0	27.427	28794.394
DMU_5_	9.507	0	0	60932.134
DMU_6_	0	0	140.289	7437.062
DMU_7_	0	21.801	27.641	9510.629
DMU_8_	3.436	4.305	0	54662.091
DMU_9_	3.113	10.770	23.777	54258.447
DMU_10_	17.497	19.333	6.550	33208.560
DMU_11_	0	0	30.015	12066.917
DMU_j_	Confidence level (*γ* = 0.75)			
	$\rho_{1}^{*}$	$\rho_{2}^{*}$	$\varphi_{1}^{*}$	$\varphi_{2}^{*}$
DMU_3_	0	3.830	27.805	45413.861
DMU_4_	0.803	0	21.235	22034.066
DMU_5_	9.738	0	0	56911.740
DMU_6_	0	0	133.327	5265.369
DMU_7_	0	19.750	25.907	8427.328
DMU_8_	4.766	0	0	51680.884
DMU_9_	2.670	10.160	19.170	53997.170
DMU_10_	16.75	19	3.330	32759.840
DMU_11_	0	0	8.649	5769.371

In Table 12, the second and third columns show the maximum decrease in the total number of computers and the number of staff for inefficient branches, respectively, while maintaining their inefficiency. Similarly, the fourth and fifth columns show the maximum increase in the sum of insured individuals’ contracts and all individuals under insurance for inefficient branches while keeping their inefficiency. For instance, consider DMU_10_ with the confidence level of *γ* = 0.25 in Table 12. DMU_10_ = (*x*_*110*_, *x*_*210*_, *y*_*110*_, *y*_*210*_) remains inefficient if $\tilde{DMU_{10}}=\left(x_{110}-{\overset{⌢}{\rho }}_{1},\;x_{210}-{\overset{⌢}{\rho }}_{2},\;y_{110}+{\overset{⌢}{\varphi }}_{1},\;y_{210}+{\overset{⌢}{\varphi }}_{2}\right)$ in which, $0\leq {\overset{⌢}{\rho }}_{1}\leq 18.243$, $0\leq {\overset{⌢}{\rho }}_{2}\leq 19.667$, $0\leq {\overset{⌢}{\varphi }}_{1}\leq 9.770$ and $0\leq {\overset{⌢}{\varphi }}_{2}\leq 33657.280$. That means if the number of computers in Branch 10decreases by a maximum of 18.243,and the number of staff decreases by a maximum of 19.667, as well as if the sum of insured individuals’ contracts increases by a maximum of 9.770 and all individuals under insurance increase by a maximum of 33657.280, then Branch 10 will remain inefficient.

Now, by considering the adjustment parameter of 0.25, we estimated the performance of 12 branches of the Social Security Organization for some confidence levels in the case of *γ* ≤ 0.25. The evaluation results are documented in Table 13.

**Table 13:** Evaluation of 12 DMUs in the case of *γ* ≥ 0.25.

DMU_j_	Confidence levels (*γ* ≥ 0.25)							
	*γ* = 0.25		*γ* = 0.5		*γ* = 0.75		*γ* = 1	
	Z*	Efficiency	Z*	Efficiency	Z*	Efficiency	Z*	Efficiency
DMU_1_	0	Efficient	0	Efficient	0	Efficient	0	Efficient
DMU_2_	0	Efficient	0	Efficient	0	Efficient	0	Efficient
DMU_3_	33003.526	Inefficient	29152.963	Inefficient	24039.227	Inefficient	0	Efficient
DMU_4_	0	Efficient	0	Efficient	0	Efficient	0	Efficient
DMU_5_	0	Efficient	0	Efficient	0	Efficient	0	Efficient
DMU_6_	0	Efficient	0	Efficient	0	Efficient	0	Efficient
DMU_7_	0	Efficient	0	Efficient	0	Efficient	0	Efficient
DMU_8_	11163.993	Inefficient	0	Efficient	0	Efficient	0	Efficient
DMU_9_	48197.830	Inefficient	43148.673	Inefficient	44598.669	Inefficient	0	Efficient
DMU_10_	25554.342	Inefficient	21293.539	Inefficient	15092.465	Inefficient	5095.558	Inefficient
DMU_11_	0	Efficient	0	Efficient	0	Efficient	0	Efficient
DMU_12_	0	Efficient	0	Efficient	0	Efficient	0	Efficient

As shown in Table 13, all of the branches are efficient in various confidence levels except Branch 3, Branch 8, and Branch 9 which are inefficient in some confidence levels, also Branch 10 which is inefficient in all the confidence levels.

Tables 14 and 15 document the stability radius for efficient branches and inefficient branches respectively, with various confidence levels of *γ* ≥ 0.25.

**Table 14:** Sensitivity analysis for efficient DMUs in the case of *γ* ≥ 0.25.

DMU_j_	Confidence level (*γ* = 0.25)			
	$\rho_{1}^{*}$	$\rho_{2}^{*}$	$\varphi_{1}^{*}$	$\varphi_{2}^{*}$
DMU_1_	8.923	0	42.627	0
DMU_2_	0	27.250	0	3764
DMU_4_	0	3.147	0	0
DMU_5_	0	16.080	74.660	0
DMU_6_	2.611	0	0	0
DMU_7_	0.338	0	0	0
DMU_11_	2.257	4.461	0	0
DMU_12_	13.814	1.243	145.947	0
DMU_j_	Confidence level (*γ* = 0.5)			
	$\rho_{1}^{*}$	$\rho_{2}^{*}$	$\varphi_{1}^{*}$	$\varphi_{2}^{*}$
DMU_1_	9.227	0	51.475	0
DMU_2_	0	28.167	0	4126.333
DMU_4_	0	4.610	0	0
DMU_5_	0	17.050	131.103	0
DMU_6_	3.227	0	0	0
DMU_7_	1.339	0	0	0
DMU_8_	0	3.277	3.550	0
DMU_11_	2.165	6.152	0	0
DMU_12_	14.019	1.971	163.915	0
DMU_j_	Confidence level (*γ* = 0.75)			
	$\rho_{1}^{*}$	$\rho_{2}^{*}$	$\varphi_{1}^{*}$	$\varphi_{2}^{*}$
DMU_1_	9.531	0	60.089	0
DMU_2_	0	29.083	0	4488.667
DMU_4_	0	6.276	0	0
DMU_5_	0	18.020	187.547	0
DMU_6_	3.844	0	0	0
DMU_7_	2.743	0	0	218.667
DMU_8_	0	4.133	25.770	0
DMU_11_	1.885	8.165	0	0
DMU_12_	14.222	2.704	181.610	0
DMU_j_	Confidence level (*γ* = 1)			
	$\rho_{1}^{*}$	$\rho_{2}^{*}$	$\varphi_{1}^{*}$	$\varphi_{2}^{*}$
DMU_1_	9.835	0	68.475	0
DMU_2_	0	30	0	4851
DMU_3_	0	0.350	0	0
DMU_4_	0	8.295	0	0
DMU_5_	0	18.990	243.990	0
DMU_6_	3.642	1.603	0	0
DMU_7_	2.990	0	0	1395
DMU_8_	0	4.789	48.811	0
DMU_9_	0	2.191	0	0
DMU_11_	1.326	10.658	0	0
DMU_12_	14.423	3.443	199.037	0

**Table 15:** Sensitivity analysis for inefficient DMUs in the case of *γ* ≥ 0.25.

DMU_j_	Confidence level (*γ* = 0.25)			
	$\rho_{1}^{*}$	$\rho_{2}^{*}$	$\varphi_{1}^{*}$	$\varphi_{2}^{*}$
DMU_3_	0	0	6.339	32997.187
DMU_8_	9.473	0	0	11154.520
DMU_9_	1.970	0	0	48195.860
DMU_10_	16.832	0	0	25537.510
DMU_j_	Confidence level (*γ* = 0.5)			
	$\rho_{1}^{*}$	$\rho_{2}^{*}$	$\varphi_{1}^{*}$	$\varphi_{2}^{*}$
DMU_3_	0	0	2.289	29150.674
DMU_9_	2.822	0	0	43145.851
DMU_10_	17.363	0	0	21276.176
DMU_j_	Confidence level (*γ* = 0.75)			
	$\rho_{1}^{*}$	$\rho_{2}^{*}$	$\varphi_{1}^{*}$	$\varphi_{2}^{*}$
DMU_3_	0.155	0	0	24039.072
DMU_9_	3.255	14.418	0	44580.997
DMU_10_	18.525	0	0	15073.940
DMU_j_	Confidence level (*γ* = 1)			
	$\rho_{1}^{*}$	$\rho_{2}^{*}$	$\varphi_{1}^{*}$	$\varphi_{2}^{*}$
DMU_10_	20.903	0	0	5074.655

The interpretation of the last two tables is similar to that of Tables 11 and 12. Also, from the comparison between Tables 10 and 13, we find that in the case of *γ* > *η*, the number of efficient DMUs is greater than in the case of *γ* < *η*. Also, as the level of confidence increases, DMUs approach the efficient frontier and the number of efficient DMUs increases.

Considering the discriminatory power of the *GF* measure compared to classical fuzzy methods, decision-makers and managers can achieve their desired results by including their preferences on the optimistic-pessimistic parameter at any confidence level in the fuzzy environment. As a result,decision-makers will be able to be aware of all efficiency scores and efficiency stability based on different optimistic-pessimistic attitudes and confidence levels. Thus, the *GF* measure provides more comprehensive information and is very proper for real-life decision-making problems.

**Fig 1:**
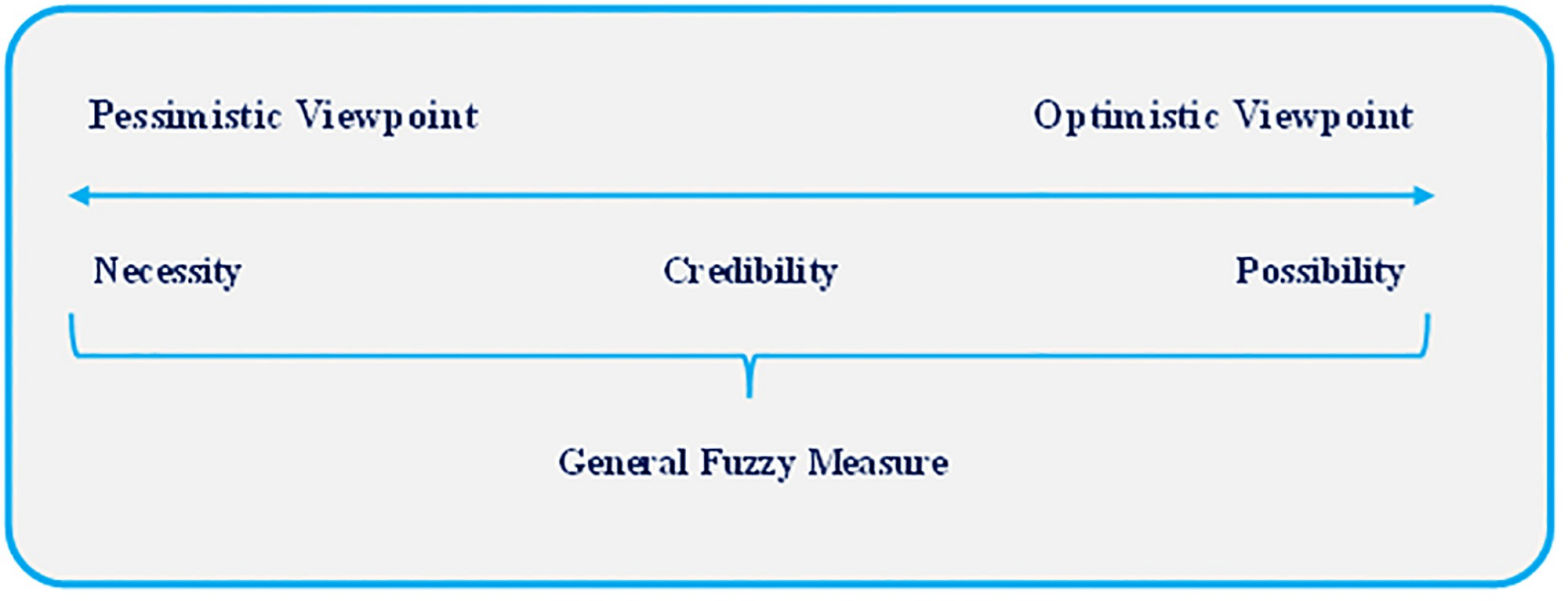
The viewpoint of fuzzy measures.

Notably, it is worth noting that the measure of the *GF* converts into the measures of *Pos*, *Nec*, and *Cr* if *η* is equal to100%, 0%, and 50%, respectively (Xu and Zhou [59]). Fig 1 is taken from Peykani et al. [61] to illustrate the attitude of fuzzy measures of *Pos*, *Nec*, *Cr*, and *GF*.

As shown in Fig 1, in the *GF* measure only by adjusting *η*, decision-makers customize their preferences.

For evaluating the efficiency sensitivity analysis of organizations, the consideration of input and output changes is very significant. The proposed fuzzy sensitivity analysis approach could be used as a “what-if” tool in managers’ strategies to improve their managerial performance. In the sense that management is able to know what may happen to the efficiency of units if input or output data is altered. As a result, this subject can lead managers to better decision-making. Furthermore, this technique can be applied to each application of real-world problems such as hospitals, universities, schools, banks, companies, etc., since these rates of change give useful information to managers or decision-makers from the managerial and economic perspectives.

## 5. Conclusion

This research seeks to investigate the sensitivity and stability of efficiency for efficient and inefficient DMUs in the additive DEA model in the presence of fuzzy data so that by identifying the permitted changes in the data, the efficiency classification of the DMUs remains unchanged. In order to tackle the uncertainty of fuzzy chance constraints in the fuzzy DEA sensitivity analysis model and convert them into their equivalent crisp values, the General Fuzzy (*GF*) approach and chance-constrained programming are used. The *GF* approach, which incorporates all prior fuzzy DEA techniques based on possibility (an optimistic viewpoint), necessity (a pessimistic viewpoint), and credibility (a compromise viewpoint), is an adjustable and flexible fuzzy DEA strategy. The previous three measures are formulated in three separate models while the *GF* measure is taken into account by setting an optimistic-pessimistic parameter in one model so that, by altering this parameter and according to different confidence levels, decision-makers can obtain their desired results.

For future studies, the proposed approach can be used as a suitable framework for evaluating performance and analyzing efficiency sustainability in various fields of management and engineering, such as supply chain, transportation, energy, stock market, mutual fund, hotel, etc. [65–69]. Also, to tackle data uncertainty, Z-number theory [70, 71], and uncertainty theory [72, 73] can be considered.
